# Reducing family and school-based violence at scale: a large-scale pre–post study of a parenting programme delivered to families with adolescent girls in Tanzania

**DOI:** 10.1136/bmjgh-2024-015472

**Published:** 2024-11-24

**Authors:** Jamie Lachman, Joyce Wamoyi, Mackenzie Martin, Qing Han, Francisco Antonio Calderón Alfaro, Samwel Mgunga, Esther Nydetabura, Nyasha Manjengenja, Mwita Wambura, Yulia Shenderovich

**Affiliations:** 1 Department of Social Policy and Intervention, University of Oxford, Oxford, UK; 2 MRC Social and Public Health Sciences Unit, University of Glasgow, Glasgow, UK; 3 Parenting for Lifelong Health, Oxford, UK; 4 Centre for Social Science Research, University of Cape Town, Cape Town, South Africa; 5 National Institute for Medical Research Mwanza Research Centre, Mwanza, Mwanza, Tanzania, United Republic of; 6 Department of Psychiatry and Behavioural Neurosciences, McMaster University, Hamilton, Ontario, Canada; 7 School of Public Health, University of Alberta, Edmonton, Ontario, Canada; 8 Department of Sexual and Reproductive Health, National Institute for Medical Research Mwanza Research Centre, Mwanza, Tanzania, United Republic of; 9 Pact Tanzania, Dar es Salaam, Tanzania, United Republic of; 10 Clowns Without Borders South Africa, Pietermaritzburg, South Africa; 11 Centre for the Development and Evaluation of Complex Interventions for Public Health Improvement (DECIPHer), School of Social Sciences, Cardiff University, Cardiff, UK

**Keywords:** Global Health, Child health, Cohort study, Intervention study

## Abstract

**Background:**

Parenting programmes, including those delivered in the Global South, are effective strategies to reduce violence against children (VAC). However, there is limited evidence of their impact when implemented at scale within routine delivery systems. This study aimed to address this gap by evaluating the real-world delivery of Parenting for Lifelong Health for Teens in Tanzania.

**Methods:**

Participating parents/caregivers and their adolescent girls were recruited by local implementing partners in 2020–2021 as part of a community-based HIV prevention initiative focused on addressing drivers of female adolescent HIV-vulnerability such as VAC, caregiver–adolescent relationships and sexual reproductive health communication. The 14-session, group-based parenting programme was delivered by trained teachers and community facilitators. Quantitative surveys administered by providers measured a variety of outcomes including child maltreatment (primary outcome) and multiple secondary outcomes linked to increased risk of VAC. Multilevel models examined pre–post effects as well as variation by attendance and baseline demographic variables.

**Results:**

Pre–post data from 27 319 parent/caregiver–child dyads were analysed, of which 34.4% of parents/caregivers were male. Analyses showed large reductions in child maltreatment (parents/caregivers: IRR=0.55, (95% CI 0.54, 0.56); adolescents: IRR=0.57, (95% CI 0.56, 0.58)), reduced intimate partner violence experience, reduced school-based violence, increased communication about sexual health, reduced poor supervision, reduced financial insecurity, reduced parenting stress, reduced parent and adolescent depression, and reduced adolescent conduct problems. In contrast to these positive outcomes, parents/caregivers and adolescents also reported reduced parental positive involvement and support of education, with those experiencing greater adversity reporting less change than those with less adversity.

**Conclusions:**

This study is the first to examine the large-scale implementation of an evidence-based parenting programme in the Global South. Although additional research is necessary to examine potential negative effects on positive parenting and parent support of education, findings suggest that Furaha Teens can sustain its impact on key outcomes associated with VAC when delivered at scale.

**WHAT IS ALREADY KNOWN ON THIS TOPIC**

- Violence against children (VAC) is a prevalent public health issue with serious short-term and long-term consequences. Over 72% of children in Tanzania report experiencing physical violence by age 18. Caregivers, relatives and teachers are the most common perpetrators, with corporal punishment considered normative. While there is substantial global evidence that parenting programmes have been successful in reducing VAC and that they have the potential to support large numbers of families if delivered widely, few have been evaluated when implemented as part of routine services and almost none at scale—especially those delivered to families with adolescent girls.

**WHAT THIS STUDY ADDS**

- This study is the first to use a non-randomised design to evaluate the pre–post changes of an evidence-based parenting programme delivered at scale as part of routine services in a low-income and middle-income country. Conducted within a broader community-based HIV prevention initiative targeting families with adolescent girls, it significantly contributes to our understanding of how such programmes can reduce VAC—a major factor driving HIV incidence among adolescent girls. Additionally, it highlights the challenges of relying on implementing agencies to collect monitoring and evaluation data during large-scale delivery.

**HOW THIS STUDY MIGHT AFFECT RESEARCH, PRACTICE OR POLICY**

- Results showing reductions in caregiver-reported and adolescent-reported physical and emotional maltreatment suggest that the programme has sustained impacts beyond initial randomised controlled trial testing. Reported reductions in intimate partner violence by female parents/caregivers and school-based violence by adolescent girls suggest wider potential impacts of a programme primarily focused on parent–child relationships. Programmes may need to be embedded in a wider range of services with structured referral processes for more vulnerable families who reported less positive change than those who were less vulnerable.

## Introduction

 Over one billion children experience some form of violence each year with higher rates in low-income and middle-income countries (LMICs).^1^ Recent meta-analyses estimate a 42.8% prevalence of child victimisation of physical domestic and family violence in West Asia and Africa.^2^ The immediate and long-term health impacts of violence against children (VAC) are well documented,^3^ with an annual global cost of non-fatal domestic child abuse estimated at almost US$3.6 trillion or 4.2% of the world gross domestic product.^4^ Given these financial and societal costs, prevention of VAC has become a global health priority as Sustainable Development Goal Target (SDG) 16.2.^5^

In Tanzania, 72% of individuals aged 13–24 reported experiencing violence before age 18 in a survey conducted in 2009.^6^ A study in 2014 with 409 children aged 6–15 found corporal punishment rates of 51% in the past year and 95% over the lifetime.^7^ The alarming rates of VAC in Tanzania extend beyond the home with studies finding teacher-reported violence of 95% and 74% of students reporting use of caning in schools.^8^ As a result, the Tanzanian government has prioritised ending VAC as part of a National Plan of Action to End Violence against Women and Children which includes parenting as one of eight key prevention strategies.^9^

Randomised controlled trials (RCTs) of parenting programmes have consistently shown reductions in VAC. A recent systematic review commissioned by the WHO included 131 RCTs of parenting programmes showing reduced child maltreatment (k=20, d=−0.39, 95% CI −0.61 to –0.17) and harsh parenting (k=58, d=−0.47, 95% CI −0.61 to –0.32) along with increased positive parenting and reduced parent mental health problems.^10^

Despite increasing evidence indicating the positive impacts of parenting programmes to prevent VAC, there is very limited research on their implementation and impact at scale.^11^ Large-scale delivery of parenting programmes in high-income countries has shown mixed results (eg, Triple P in North Carolina, USA and in Glasgow, UK).^12 13^ In Peru, the Cunas Mas parenting programme showed improvements in child development when delivered to 67 000 caregivers of children ages 0–36 months but did not measure VAC outcomes.^14^ There is also limited evidence on the effectiveness of parenting interventions delivered to male caregivers who are often under-represented in such programmes.^15^ Moreover, conducting large-scale RCTs is often cost-prohibitive or not feasible in low-resource settings.^16^ While lacking the ability to draw causal assumptions of intervention effectiveness and often relying on often ‘flawed, uncertain, proximate and sparse’ (FUPS) monitoring and evaluation data,^17^ non-randomised pre–post studies embedded within routine service delivery systems may provide an opportunity to further understand the impact of these programmes at large scale.^11^

## Parenting for Lifelong Health and the Furaha Teens Programme in Tanzania

The Parenting for Lifelong Health (PLH) programme for parents/caregivers and their adolescents (PLH-Teens) was initially developed and tested to address the link between violence against adolescent girls and the increased risk of HIV incidence in low-income families in South Africa.^18 19^ Results from a cluster RCT of the programme involving 522 families with children aged 10–17 in South Africa demonstrated reductions in physical and emotional abuse, along with improvements in positive parenting, parental supervision, caregiver mental health and other secondary outcomes.^20^ Following these positive findings and the established connections between VAC and HIV incidence among adolescent girls, PLH-Teens was designated by US President’s Emergency Plan for AIDS Relief as an approved evidence-based programme for the DREAMS Initiative (Determined, Resilient, Empowered, AIDS-free, Mentored and Safe).

The DREAMS Initiative aims to reduce HIV-infection among adolescent girls and young women in sub-Saharan Africa by addressing structural drivers for increased HIV risk through a core package of evidence-based interventions, including parenting programmes delivered to families with adolescent girls.^21^ The inclusion of PLH-Teens as an approved DREAMS Initiative programme contributed to its rapid dissemination to more than 20 HIV-priority countries, primarily in sub-Saharan Africa.^22^ This included its adaptation for families with in-school adolescent girls aged 10–14 as part of a DREAMS Initiative in Tanzania called the Kizazi Kipya (‘New Generation’) Project. As part of the Kizazi Kipya Project, Pact Tanzania—a Tanzania-based non-government organisation (NGO) that is part of Pact Global, an international NGO with almost 40 country offices worldwide—delivered PLH-Teens (locally known as the Furaha Caring Programme for Parents and Teens, or Furaha Teens) to 30 642 caregivers and 44 447 adolescent girls from June 2016 to December 2021.^23^

## Furaha Adolescent Implementation Research Study

The Furaha Adolescent Implementation Research (FAIR) study used a non-randomised design to examine the impact of the Furaha Teens programme during its scale-up in Tanzania from 2020 to 2021.^24^ Embedded within the Kizazi Kipya Project,^24^ this study used routine monitoring and evaluation data collected by the implementing partner to address the following research questions: (1) What is the quality of outcome data collected at scale in terms of completeness and measurement reliability? (2) Are there pre–post changes in child maltreatment and other behavioural and psychological outcomes linked to VAC as reported by adolescent girls and their parents/caregivers? (3) How are baseline characteristics and implementation variables associated with pre–post changes in behaviour and psychological outcomes and if so, how?

## Methods

### Setting

The implementation of Furaha Teens and data collection was conducted by Pact Tanzania between February 2020 and February 2021 in eight rural and semiurban districts where the full Kizazi Kipya intervention package was being delivered for families with in-school adolescent girls aged 10–14. The protocol of the full study was published a priori.^24^ The current paper focuses on the pre–post changes observed using secondary data from 36 679 adolescent girls and 33 728 parents/caregivers (a total of 70 407 beneficiaries). This manuscript followed the guidelines for reporting on non-randomised trials.^25^

### Participants

Adolescents and their parents/caregivers who participated in Furaha Teens were invited by Pact Tanzania to partake in the study on enrolment in the Kizazi Kipya Project. Adolescent girls had to be 10–14 years old, live in the same household as their parents/caregivers at least 4 days a week in the past month, have consent from their primary caregiver and provide assent. Parents/caregivers needed to be at least 18 years or older, responsible for the well-being of the indicated adolescent girl and provide consent to participate. Only one primary caregiver and one child were selected to participate in the Furaha Teens programme per household. If there were more than one caregiver per household, caregivers were asked to decide which one would participate based on their daily interaction with children and availability to participate in the programme.

## Programme delivery

Furaha Teens is the localised version of PLH-Teens, a 14-session, group-based parenting programme that aims to strengthen parents/caregivers’ skills by using positive parenting and relationship-building techniques. Additional material for HIV prevention was added in 2018 in accordance with USAID funding requirements. All programme materials were translated into Kiswahili, licensed under a Creative Commons Attribution Share Alike 4.0 licence and freely available online (www.parentingforlifelonghealth.org).

Local teachers and community volunteers (N=444) received 30 hours of facilitator training provided by PLH trainers from Clowns Without Borders South Africa, a South Africa-based NGO that supports the dissemination of PLH programmes. The facilitators delivered 2–3.5 hours group sessions to parents/caregivers and their adolescent girls (20 families per group, 40 participants in total). 10 sessions were delivered to joint groups of parents/caregivers and their adolescents, and 4 were delivered to parents/caregivers and adolescents separately. Facilitators used a participatory approach grounded in social learning theory to support caregiver–adolescent relationship building and skills acquisition with group discussions, role-plays and home activity assignments.^20^ Participants who missed group sessions were followed up with home visits, although whether a session was delivered to a family via groups or home visits was not captured by attendance registers (see online supplemental table 1 for Template for Intervention Description and Replication (TiDieR) checklist description of Furaha Teens).

Although the overall implementation of Furaha Teens was coordinated by Pact Tanzania, five subcontracted local implementing partners were responsible for direct programme delivery across the eight districts. Due to the limited number of families each implementing partner could reach at one time, the scaled delivery of Furaha Teens was implemented over three successive waves (wave 1: January–July 2020; wave 2: July–October 2020; wave 3: December 2020–March 2021). Furthermore, in-person delivery was paused during wave 1 from April to June 2020 because of movement restrictions during COVID-19. Finally, although the sessions were intended to be delivered over 14 weekly sessions, programme delivery varied with some groups delivering two sessions per week.

### Outcomes

All measures were open-access, freely available and translated into Kiswahili with back-translation for accuracy. Given limited capacity of implementation staff to administer complete measurements to thousands of participants during programme delivery, item response theory was used to create shorter scales by identifying items most representative of the underlying constructs of original measurements.^22^

The primary outcome was parent/caregiver-reported and adolescent-reported child maltreatment (four items), with subscales for physical and emotional abuse (two items each) from the International Society for the Prevention of Child Abuse and Neglect Child Abuse Screening Tools-Trial Version.^26^ Secondary outcomes included parent-caregiver-reported and adolescent-reported positive parental involvement and poor supervision, child behaviour problems, parenting stress, parent/caregiver and adolescent endorsement of corporal punishment, parent/caregiver and adolescent depression, parental support of education, financial insecurity, and sexual health communication. We also assessed intimate partner violence experience (female parent/caregiver report) and perpetration (male parent/caregiver report), as well as adolescent-reported experience of school bullying (see table 1).

**Table 1:** Summary of outcome measures assessed

Outcome	Measurement	Source	Items
Child maltreatment	ICAST-Trial	Adult and adolescent	4
Child physical abuse	ICAST-Trial	Adult and adolescent	2
Child emotional abuse	ICAST-Trial	Adult and adolescent	2
Positive parental involvement	Alabama Parenting Questionnaire	Adult and adolescent	3
Poor supervision	Alabama Parenting Questionnaire	Adult and adolescent	3
Endorsement of corporal punishment	UNICEF Multiple Indicator Cluster Survey	Adult and adolescent	1
Child behaviour problems	Strengths and Difficulties Questionnaire-Conduct Problems	Adult and adolescent	5
Parenting stress	Parenting Stress Scale	Adult	2
Depression	Centre for Epidemiologic Studies Depression Scale	Adult and adolescent	3
Parental support of education	Parental Support for School Scale	Adult and adolescent	2
Financial insecurity	Family Financial Coping Scale	Adult and adolescent	2
Sexual health communication	Risk Avoidance Planning Scale	Adult and adolescent	3
Intimate partner violence-experience	Conflict Tactics Scale-Short Form	Female adult	2
Intimate partner violence-perpetration	Conflict Tactics Scale-Short Form	Male adult	2
Experience of school bullying	Locally developed	Adolescent	3

ICAST, International Society for the Prevention of Child Abuse and Neglect Child Abuse Screening Tools-Trial Version.

The parent involvement and parent support of education items were not included in wave 1 due to an error in which the implementing partner omitted the final page of the paper-based surveys which included these scales. This omission was identified by Pact Tanzania monitoring and evaluation teams, and the items were added for subsequent waves.

Basic demographic variables were chosen from the UNICEF Multiple Indicator Cluster Survey and included parent/caregiver and adolescent age, parent gender, adolescent school enrolment and highest level of education attained, parent/caregiver employment, parent/caregiver relationship status, parent/caregiver biological relationship to adolescent, and parent/caregiver and adolescent literacy. The following variables linked to increased risk of VAC were assessed at baseline: adolescent orphan status, adolescent parenthood, family financial insecurity and the presence of an unwell adult or child, alcohol or drug use, family conflict, recent experience of tuberculosis or AIDS-related death, or a child with disabilities in the household.^27^ We also included type of facilitator (teacher/volunteer) and programme delivery location (community-only/school and community) as potential factors associated with intervention effects.

### Data collection and cleaning

Participants provided consent to contribute their data to the study and were enrolled in the Furaha Teens programme. Community facilitators used paper forms to collect data. During each wave of implementation, baseline data were collected at the beginning of the first group session and post-test data were collected during the last programme session. Data from paper forms were entered manually into CommCare by data clerks employed by Pact Tanzania based in implementation sites. Pact Tanzania conducted random spot checks of data entry at implementation sites. Fully anonymised data were uploaded by Pact Tanzania to a password protected and encrypted server hosted in Tanzania with data backed up in the UK. Data were then cleaned by research staff based in the UK with support provided by Tanzanian research and implementation staff.

### Data analysis

**Figure 1:**
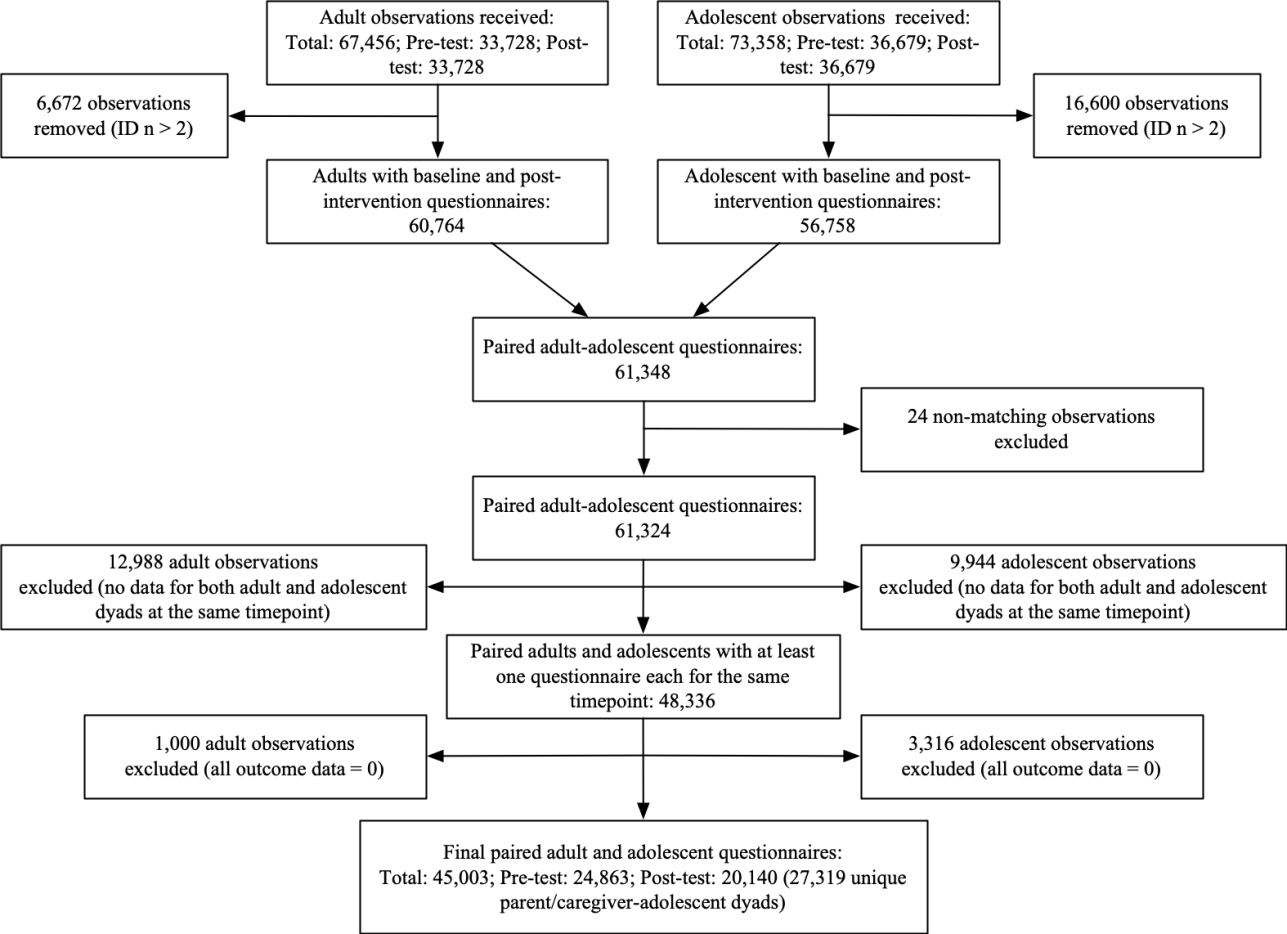
Data cleaning flow chart.

Data were cleaned and analysed in R. Reliability of scales with at least three items was examined using Cronbach’s alpha (α) and omega (ω), and Pearson correlations for scales with only two items. Intraclass correlation coefficients (ICCs) were calculated to determine which potential random effect factors (ie, participant IDs, facilitator IDs and wave of implementation) could be included in subsequent multilevel regression models. Distribution tests were used to determine the most appropriate method for analysing frequency scales (online supplemental figure 1), with all indicating a Poisson distribution (online supplemental tables 2 and 3). As a result, multilevel Poisson/linear regressions were used to test pre–post differences in primary and secondary outcomes. In all analyses, the adolescent-reported or parent/caregiver-reported outcome was the dependent variable, the pretest and post-test time point was the fixed effect, and the participant ID was the random effect. Furthermore, a sensitivity analysis was conducted to assess the potential influence of unmeasured confounding factors that might render an observed intervention effect ineffective. We employed the E-value estimation method to measure the minimum level of association strength necessary for an unmeasured confounder with both the exposure (intervention or non-intervention) and the outcome (behavioural outcomes) to potentially undermine the observed connection between exposure and outcome.^28^ We also conducted sensitivity analyses to determine whether study attrition had an influence on intervention outcomes. This involved only removing duplicate data (ie, step 1 in the data cleaning process as shown in figure 1) which resulted in a dataset with 30 382 caregivers and 28 379 adolescents.

We conducted additional analyses to further understand whether intervention effects on child maltreatment varied by parent/caregiver, adolescent, household or implementation characteristics. Analyses involved include an interaction term of time point and potential predictors to each Poisson/linear regression model. Due to the large sample size and large number of analyses conducted, the magnitude of effect was used to identify meaningful effects rather than significance tests.^29^ Thresholds for small, medium and large effects were developed a priori to allow for interpretation of different effects sizes within the study. Incidence rate ratios (IRRs) differing by more than 10% were considered a small effect (0.80≤IRR<0.90 or inversely 1.25≤IRR<1.11), more than 20% a medium effect (0.70≤IRR<0.80 or inversely 1.43≥IRR>1.25) and more than 30% as a large effect (IRR<0.70 or inversely IRR>1.43).

## Results

### Data quality

Several steps were taken for data cleaning. First, 6672 parents/caregivers and 16 600 adolescents were removed from the raw data due to duplicate measurements. Second, parents/caregivers and adolescents were matched into pairs using family IDs from their data. There were 24 instances in which caregivers or adolescents matched with multiple counterparts, which were further removed. Third, we ensured that data for both parents/caregivers and adolescents from the same family were available at the same time point (either pretest or post-test); otherwise, they were also removed. To be noted, the numbers of pretest and post-test records in the raw data were the same due to the data format conversion from wide form to long form, during which any missing record at pre or post-test were temporarily labelled, ‘not available’. In this third step, those missing records were identified and removed. There were 12 988 instances where parent/caregiver-adolescent pairs did not have data at the same time point and were thus removed from the data. This data cleaning step could result in different numbers for pretest and post-test. For example, if there were data for both caregiver and adolescent at pretest but only data for the parent/caregiver at post-test, only the pretest pair was retained. Fourth, to further ensure data quality, observations, where participants marked zero (or the first option) for every item in the questionnaire, were removed, as these data were deemed potentially insincere or invalid. Consequently, due to invalid parent/caregiver questionnaires, 1000 parent/caregiver-adolescent pairs were removed, and then based on invalid adolescent questionnaires, 2333 parent/caregiver-adolescent pairs were further removed. The final dataset used for primary analysis included 45 003 paired parent/caregiver-adolescent surveys, with 24 863 at pretest and 20 140 at post-test, resulting in 27 319 parent–child dyads (figure 1).

### Outcome reliability and intracluster corelations

Analyses of most scales showed high reliability (online supplemental table 4). Scales measuring physical or emotional abuse include items capturing different types of abuse (eg, spanking vs hitting with a stick) could explain the lower correlations observed on these scales. Participant IDs had a relatively large ICC of 0.70 and were thus incorporated as a random effect in the subsequent regression models. Facilitator ID also had a large ICC of 0.75, this variable had a high level of missingness in the dataset (56.3% missing values) and was not included as a random effect. Wave of implementation had a very small ICC of 0.12 and was also not included as a random effect (online supplemental table 5).

## Demographics

Descriptive characteristics of the sample are summarised in table 2. More than one-third of the parents/caregivers were male (34.4%), which was high given that parenting programmes typically struggle to engage men.^30^ The mean age of parents/caregivers was 44.11 years (SD=11.82), with men, on average, being older than women. Most parents/caregivers indicated that they were partnered (85.4%), the biological parent of the adolescent (86.3%) and could read (60.3%). More than two-thirds were unemployed (69.8%) and just over half struggled to buy food and essentials in the previous month (52.3%). One in 10 reported an adult who was unwell living in the house, 6.9% reported being affected by tuberculosis or HIV/AIDS, 14.4% reported issues related to alcohol or drugs in the household, 11.5% reported family conflict, 11.6% reported having an unwell child living in the house, 6.4% reported having a child with some form of disability and 18.2% reported that their child was either a single or double orphan. As expected, all adolescent respondents were female. Although the inclusion criteria in the protocol required adolescent girls to be 10–14 years, the actual age range was 9–16 years (M=11.64, SD=1.56) due to variations in programme delivery across the sample. Almost 4% of adolescents reported having their own child (3.8%). Over three-quarters of adolescents were able to read (77.3%) and were enrolled in school (78.2%), with an average education level between standard 4 or standard 5 (M=4.82, SD=1.85) (see table 2).

**Table 2:** Demographic characteristics of caregivers and adolescents at baseline

	Parent/caregivers n=24 863	Adolescents n=24 863
Age, M (SD)	44.11 (11.82)	11.64 (1.52)
Gender:female, n (%)	16 067 (64.6)	–
Child education level, M (SD)	4.83 (1.86)	–
Child enrolled in school, n (%)	8498 (78.2)	–
Currently employed, n (%)	7503 (30.2)	–
Marital status: partnered, n (%)	9273 (85.4)	–
Child biological son/daughter, n (%)	21 432 (86.2)	–
Household struggles to buy food or essentials, n (%)	13 005 (52.3)	10 402 (41.8)
Unwell adult in house, n (%)	2475 (10.5)	2000 (8.4)
Household affected by TB or HIV/AIDS, n (%)	1725 (6.9)	1199 (4.8)
Household affected by alcohol or drugs, n (%)	3592 (14.4)	3562 (14.3)
Household affected by arguments, n (%)	2857 (11.5)	2876 (11.6)
Unwell child in house, n (%)	2886 (11.6)	2443 (9.8)
Disability affects a child in house, n (%)	1587 (6.4)	948 (3.8)
Biological parent lives in house, n (%)	20 452 (82.3)	21 418 (86.1)
Can read easily, n (%)	14 988 (60.3)	19 218 (77.3)
Child single or double orphan, n (%)	4530 (18.2)	3764 (15.1)
Adolescent parenthood, n (%)	–	950 (3.8)
Sessions attended, n (%)	12.75 (1.84)	12.76 (1.83)
Attended all 14 sessions, n (%)	14 684 (60.3)	14 718 (60.5)
Delivery via group sessions, n (%)	22 625 (91.0)	
Location implemented: school, n (%)	8181 (77.8)	
Facilitator type: teacher, n (%)	7588 (72.1)	

All adolescents are female

TB, tuberculosis.

### Programme delivery

Parent/caregiver and adolescent programme participation was high. The average participant received 91% of programme sessions via group sessions or home visits. Out of 14 possible sessions, parents/caregivers received an average of 12.75 sessions (SD=1.84) and adolescents attended an average of 12.76 sessions (SD=1.83). There were 14 684 parents/caregivers and 14 718 adolescents who received all 14 sessions, accounting for 60.3% of the total number of parents/caregivers and 60.5% of the total number of adolescents.

## Main results

The multilevel regression results showed that most adult-reported outcomes significantly improved at post-test including reductions in primary outcomes: overall child maltreatment (IRR=0.55 (95% CI 0.54, 0.56)), physical abuse (IRR=0.51 (95% CI 0.50, 0.53)) and psychological abuse (IRR=0.56 (95% CI 0.55, 0.57)). Similar results were found for adolescent-reported primary outcomes: overall child maltreatment (IRR=0.57 (95% CI 0.56, 0.58)), physical abuse (IRR=0.56 (95% CI 0.55, 0.58)) and psychological abuse (IRR=0.55 (95% CI 0.54, 0.56)). Improvements were also found for parent/caregiver-reported and adolescent-reported poor supervision, financial insecurity, parenting stress, parenting depression, child conduct problems, emotional problems, child depression and sexual health communication. Notably, pre–post analyses found reduced intimate partner violence experience reported by female parents/caregivers (IRR=0.78 (95% CI 0.74, 0.81)), as well as adolescent-reported reduced school violence victimisation (IRR=0.84 (95% CI 0.82, 0.86)), even though Furaha Teens did not specifically target these outcomes. Analyses also found reduced positive involvement (parent: β=−1.21, p<0.001; adolescent: β=−0.82, p<0.001) and reduced support of education (parent: β=−0.72, p<0.001; β=−0.50, p<0.001). However, it is worth noting that these two behavioural variables were only measured during wave 2 of implementation at the height of the COVID-19 pandemic and were measured using a reversed scale from the other scales which may have been misinterpreted by respondents. No changes were found in male reported IPV perpetration (tables3  4).

**Table 3:** Multilevel regression analysis of parent/caregiver-reported outcomes

	Reliability (ω)	Mean_pre_	SD_pre_	Mean_post_	SD_post_	β	SE	P value	IRR	95% lower CI	95% upper CI
Overall maltreatment^*^	0.65	2.39	2.74	1.24	1.99	−0.60	0.01	<0.001	0.55	0.54	0.56
Physical abuse^*^	0.20	1.11	1.48	0.55	1.10	−0.66	0.01	<0.001	0.51	0.50	0.53
Psychological abuse^*^	0.24	1.28	1.60	0.69	1.15	−0.58	0.01	<0.001	0.56	0.55	0.57
IPV experience (female-only)^†^	0.57^¶¶^	1.07	1.75	0.73	1.45	−0.25	0.02	<0.001	0.78	0.74	0.81
IPV perpetration (male-only)^†^	0.58^¶¶^	0.85	0.16	0.76	1.57	0.01	0.03	0.236	1.03	0.98	1.09
Positive involvement^‡^	0.95	4.80	4.06	3.43	3.86	−1.21	0.05	<0.001	---	---	---
Poor supervision^‡^	0.81	1.32	1.97	0.82	1.56	−0.50	0.02	<0.001	---	---	---
Support of education^§^	0.90^¶¶^	5.17	2.81	4.38	2.67	−0.72	0.04	<0.001	---	---	---
Financial insecurity^¶^	0.79^¶¶^	2.93	2.18	1.98	1.97	−0.94	0.02	<0.001	---	---	---
Parenting stress^**^	0.70^¶¶^	3.34	2.58	1.89	2.25	−1.44	0.02	<0.001	---	---	---
Parenting depression^††^	0.67	3.92	1.45	3.37	1.41	−0.55	0.01	<0.001	---	---	---
Child conduct problems^‡‡^	0.75	1.71	1.76	1.47	1.78	−0.22	0.01	<0.001	---	---	---
Sexual health communication^§§^	0.91	2.28	1.98	3.89	2.31	1.62	0.02	<0.001	---	---	---

Positive involvement and support of education only measured during wave 2 of programme delivery.

* International Society for the Prevention of Child Abuse and Neglect (ISPCAN) Child Abuse Screening Tool-Trial.

† Revised Conflict Tactics Scale Short.

‡ Alabama Parenting Questionnaire.

§ Parental Support for School Scale.

¶ Family Financial Coping Scale.

** Parenting Stress Scale.

†† Centre for Epidemiological Studies-Depression Short-Form.

‡‡ Strengths and Difficulties Questionnaire Conduct Problems Subscale.

§§ Risk Avoidance Planning Scale.

¶¶ Pearson correlation used instead of Omega value for measures with only two items.

IRR, Incidence Rate Ratio.

**Table 4:** Multilevel regression analysis of adolescent-reported outcomes

	Reliability (ω)	Mean_pre_	SD_pre_	Mean_post_	SD_post_	β	SE	P value	IRR	95% lower CI	95% upper CI
Overall maltreatment^*^	0.64	2.33	2.87	1.23	2.23	−0.57	0.01	<0.001	0.57	0.56	0.58
Physical abuse^*^	0.18^§§^	1.14	1.60	0.61	1.26	−0.58	0.01	<0.001	0.56	0.55	0.58
Psychological abuse^*^	0.64^§§^	1.19	1.63	0.62	1.22	−0.60	0.01	<0.001	0.55	0.54	0.56
School violence experience^†^	0.79	2.35	3.19	1.59	2.84	−0.17	0.01	<0.001	0.84	0.82	0.86
Positive involvement^‡^	0.94	5.06	3.83	4.17	3.62	−0.82	0.05	<0.001	---	---	---
Poor supervision^‡^	0.77	1.43	1.98	0.81	1.46	−0.63	0.02	<0.001	---	---	---
Support of education^§^	0.90^§§^	2.83	2.67	2.28	2.70	−0.50	0.04	<0.001	---	---	---
Child depression^¶^	0.71	2.47	2.04	2.03	1.99	−0.43	0.02	<0.001	---	---	---
Child emotional problems^**^	0.90	1.59	1.99	1.61	2.06	0.02	0.03	0.770	---	---	---
Child conduct problems^††^	0.74	1.58	1.72	1.38	1.74	−0.17	0.01	<0.001	---	---	---
Sexual health communication^‡‡^	0.90	2.05	1.96	3.81	2.31	1.77	0.02	<0.001	---	---	---

Positive involvement and support of education only measured during wave 2 of programme delivery.

* International Society for the Prevention of Child Abuse and Neglect (ISPCAN) Child Abuse Screening Tool-Trial.

† Locally Developed School Violence Scale.

‡ Alabama Parenting Questionnaire.

§ Parental Support for School Scale.

¶ Centre for Epidemiological Studies-Depression.

** Strengths and Difficulties Questionnaire.

†† Strengths and Difficulties Questionnaire.

‡‡ Risk Avoidance Planning Scale.

§§ Pearson correlation used instead of Omega value for measures with only two items.

IRR, Incidence Rate Ratio.

## Sensitivity analyses

For the outcomes reported by parents, the sensitivity analysis did not reveal any small E-values that would warrant concern about the intervention effect being easily nullified by unmeasured confounders. As for the variables reported by children, only the E-value for child emotional problems was very close to the effect size, but no significant intervention effect was detected on this variable itself. Therefore, the results of this sensitivity analysis did not indicate the presence of noteworthy unmeasured confounding effects (online supplemental tables 6 and 7).

We also conducted sensitivity analyses to determine whether there were any differences between those whose data were included in the study and those whose data were excluded during the data cleaning process. Caregivers whose data were excluded in the analyses were more likely to have lower rates of employment and lower literacy levels, but less likely to have household struggles compared with caregivers who were included. Likewise, excluded adolescents were more likely to not be enrolled in school but have lower levels of family vulnerability (eg, household struggles to buy food or essentials, household affected by alcohol or drugs) compared with adolescents whose data were included in analyses (online supplemental table 8). Furthermore, sensitivity analyses using less rigorous exclusion criteria (ie, only removing duplicates) show results similar to the main analysis, with consistent significance levels and only slight changes in effect size (online supplemental tables 9 and 10).

### Factors associated with pre–post change in overall child maltreatment

Analyses found greater reductions in parent/caregiver-reported child maltreatment in families with female parents/caregivers, higher parent/caregiver literacy and employed parents/caregivers. Parents/caregivers also reported larger reductions in maltreatment in families with higher poverty, recent tuberculosis or AIDS-related death, alcohol or substance abuse, and family conflict (0.80≤IRR<0.90). There were also greater reductions in child maltreatment reported by parents/caregivers with adolescents enrolled in school (IRR=0.69 (95% CI 0.65, 0.73)). Parents/caregivers in a partnered relationship or those with an older adolescent reported smaller reductions in child maltreatment (IRR=1.46 (95% CI 1.37, 1.56); IRR=1.45 (95% CI 1.37, 1.52), respectively). Compared with those who received the programme in schools only, parents/caregivers reported smaller reductions in maltreatment when the programme was delivered on a community level (IRR=1.18 (95% CI 1.11, 1.26)), but greater reductions when the programme was delivered on mixture of community and school systems (IRR=0.81 (95% CI 0.76, 0.86)).

Similar results were reported by adolescents with the exception of parent/caregivers employment, child orphanhood status and child school enrolment which did not meet the a priori determined clinical threshold of clinical significance (ie, IRR<0.90 or inversely IRR>1.11). In contrast to parent/caregiver-report, adolescents in households with recent tuberculosis or AIDS-related deaths reported smaller reductions in child maltreatment (IRR=1.20 (95% CI 1.13, 1.27)). In addition, adolescents in families where their parents/caregivers were in a partnered relationship reported smaller reductions in child maltreatment (IRR=1.22 (95% CI 1.15, 1.30)). Adolescents living in households wherein one or more children had a disability or illness reported smaller reductions in child maltreatment (IRR=1.21 (95% CI 1.14, 1.29); IRR=1.37 (95% CI 1.31, 1.43), respectively). Substantially smaller reductions in child maltreatment were reported by older adolescents (IRR=1.50, (95% CI 1.42, 1.58)). Adolescents also reported smaller reductions in child maltreatment when the programme was delivered by community (IRR=1.21 (95% CI 1.14, 1.27)). There were also substantially smaller reductions in child maltreatment when the programme was delivered on a community level (IRR=1.63 (95% CI 1.52, 1.75)), but greater reductions in maltreatment when the programme was delivered via mixture of community and school levels (IRR=0.67 (95% CI 0.63, 0.72)) (table 5).

**Table 5:** Factors associated with pre–post change in overall child maltreatment

Variable	Adult report	Child report
Caregiver variables		
Parent/caregiver age (every 5 years)	1.04 (1.03,1.04)	1.02 (1.01,1.02)
Female parent/caregiver	0.89 (0.86,0.92)*	0.88 (0.85,0.91)*
Higher parent/caregiver literacy	0.89 (0.88,0.90)*	0.85 (0.84,0.86)*
Parent/caregiver employed	0.81 (0.79,0.84)*	0.98 (0.95,1.02)
Parent/caregiver in a partnered relationship	1.46 (1.37,1.56)‡	1.22 (1.15,1.30)*
Biological parent	1.07 (1.02,1.12)	1.01 (0.97,1.06)
Child variables		
Child age (every 5 years)	1.45 (1.37,1.52)‡	1.50 (1.42,1.58)‡
Child school enrolment	0.69 (0.65,0.73)‡	0.91 (0.86,0.96)
Child education level	0.99 (0.98.1.00)	0.95 (0.94,0.96)
Child literacy	0.92 (0.90,0.94)	0.85 (0.83,0.86)*
Child single or double orphanhood	0.89 (0.86,0.93)*	1.09 (1.05,1.13)
Child parenthood	1.15 (1.06,1.24)*	1.22 (1.13,1.32)*
Household variables		
Higher poverty	0.87 (0.84,0.90)*	0.89 (0.86,0.92)*
Adult illness	0.91 (0.86,0.95)	1.08 (1.03,1.14)
Child illness	0.91 (0.86,0.95)	1.37 (1.31,1.43)†
Child disability	0.90 (0.84,0.95)	1.21 (1.14,1.29)*
TB or AIDS-related death	0.85 (0.80,0.90)*	1.20 (1.13,1.27)*
Alcohol or substance use	0.83 (0.79,0.86)*	1.11 (1.07,1.16)
Family conflict	0.82 (0.79,0.86)*	1.11 (1.06,1.16)
Implementation variables		
Volunteer facilitator (vs teacher)	1.04 (0.99,1.10)	1.21 (1.14,1.27)*
Community delivery (vs school-only)	1.18 (1.11,1.26)*	1.63 (1.52,1.75)‡
Mixed community and school delivery (vs school-only)	0.81 (0.76,0.86)*	0.67 (0.63,0.72)‡

Analyses are reported as IRR with 95% CI.

* Small clinically significant effect (0.80≤IRR<0.90 or inversely 1.25≥IRR>1.11).

† Medium clinically significant effect (0.70≤IRR<0.80 or inversely 1.43≥IRR>1.25).

‡ Large clinically significant effect (IRR<0.70 or inversely IRR>1.43).

## Discussion

This study is the first of its kind to evaluate the large-scale impact of a parenting programme aimed at reducing VAC in LMICs. Despite its non-randomised design and reliance on routine monitoring and evaluation data, which often results in substantial missing data and limits causal inference, embedding this study within Kizazi Kipya Project—which delivered Furaha Teens to 70 407 beneficiaries—provided a unique opportunity to significantly advance our understanding of the impact of an evidence-based intervention delivered at scale within existing service delivery systems. The findings offer valuable insights into the sustained effects of such a programme when integrated into routine services. The findings are promising for the future scale-up of parenting programmes in LMICs considering evidence that interventions, including parenting programmes and other programmes for children and families, often have difficulty maintaining effects observed in randomised trials when delivered at scale and in routine service settings.^13 31 32^ The relatively high data completion rate of 60.5% is also noteworthy for the delivery setting, especially considering the implementation occurred during the COVID-19 pandemic.

The large reductions in parent/caregiver-reported and adolescent-reported child maltreatment from pretest to post-test are promising, an important finding in the field of translational science.^16^ Results are by-and-large consistent with findings from the original RCT of the programme in South Africa which also found similar reductions in child maltreatment when compared with a control group (parent/caregiver: IRR=0.39 (95% CI 0.28, 0.54); adolescent: IRR=0.71 (95% CI 0.51, 0.97)).^20^ The convergence of parent/caregiver and adolescent reports also provides additional confidence in the results.^33^ The findings are also encouraging given the large proportion of male parents/caregivers in the sample (35.4%), who are often excluded from parenting programmes.^34^ Similarly, analyses found positive changes in multiple outcomes linked to increased risk of VAC. This study found reductions in child behaviour problems, child and caregiver mental health problems, and family financial insecurity.^27^ Improvements in sexual health communication between parents/caregivers and their adolescents are encouraging, as evidence suggests strong linkages between sexual health communication and reduced adolescent risky sexual behaviour and sexual abuse.^35^ In addition, the reductions in experience of intimate partner violence and school violence found in this study indicate potential knock-on effects of parenting programmes on other forms of violence.^36^ This finding is also aligned with emerging research suggesting that improvements in parenting may accelerate impacts across multiple outcomes linked to a wider range of SDGs.^37^

The study also found reductions in positive parental involvement and parent support of education. It is possible that positive parent/caregiver–child interaction were affected by the COVID-19 pandemic, especially since the implementing partner only included these outcomes at wave 2 during the height of government restrictions, which included several months of school closures. Furthermore, parents/caregivers may have required additional emphasis on the positive parenting skills introduced during the first half of the programme, especially when the programme shifted focus to conflict resolution during the latter half. The intervention may also have served as a protective factor to even greater reductions in positive parenting due to the pandemic. Nonetheless, further research using a quasi-experimental design with controls is necessary to establish whether these results were due to the Furaha Teens programme or other factors.

This study was also the first of its kind to conduct analyses of factors associated with pre–post changes in child maltreatment at scale, thus allowing for greater specificity on whether there may be differential effects based on population characteristics. The large sample size also provided substantial statistical power to detect interaction effects that many smaller studies of parenting programmes lack.^38 39^ However, it is important to note that results from parent/caregiver reports or adolescent reports were somewhat contradictory. For instance, while parents/caregivers who experienced adversity reported greater reductions in child maltreatment (ie, child orphanhood, higher poverty, tuberculosis or AIDS-related death, alcohol or substance use problems, or family conflict), adolescents reported smaller reductions (ie, child illness, child disability or tuberculosis or AIDS-related death). More vulnerable adolescents may have reported less positive change during the intervention than less vulnerable adolescents due to additional stressors experienced. Yet, another explanation is that more vulnerable parents/caregivers may have engaged more in the programme due to perceptions that the programme may help them with adversities and thus these parents/caregivers may have responded more positively than those who were less vulnerable. Interestingly, parents/caregivers and adolescents in single-parent families reported greater reductions in child maltreatment when compared with those with parents/caregivers in partnered relationships.

The larger associations warrant further discussion. Families with younger children reported substantially higher reductions in maltreatment than those with older children, potentially due to lower baseline rates of maltreatment, though it could also have been due to the reduced effectiveness of the intervention. Furthermore, children enrolled in school reported much greater reductions in maltreatment than those who were not enrolled suggesting possible community-level influences, especially since those who received the programme within the schools system and by teachers showed greater effects. Moreover, as a comparison group was not available, it is possible that some subgroups would have experienced greater or smaller changes in their outcomes over time in the absence of the intervention. Finally, the thresholds for small, medium and large effect sizes were developed a priori for the purposes to allow for interpretation of relative results within this study. Future research is recommended on magnitudes of these effects, especially within the field of parenting interventions and the prevention of child maltreatment.

### Limitations of working with FUPS data in a real-world context

This study represented a unique opportunity to analyse the largest dataset known on a parenting programme delivered in a low-resource, community setting at scale. While this dataset is novel and valuable, it had limitations in terms of data quality. Restrictions during COVID-19 and challenges conducting monitoring and evaluation hindered data quality. Data collection via paper forms is commonly used within this context due to limited access to technology in the field. However, this process is prone to errors in the form of misplacing forms and entering the data incorrectly. In addition, the reliance on abbreviated measurements that were not psychometrically tested may have resulted in imprecise assessments of outcomes. Most of the measures had acceptable levels of reliability (see tables4  5 and online supplemental table 4) with low correlations for child maltreatment and IPV measures expected due to different individual-level behaviours (eg, caregivers may consistently use one form of physical discipline over another). Although the implementing partner did not have the capacity to administer longer assessments using complete scales, an alternative approach randomly selecting participants from the wider sample may have provided more robust results.

There were also a variety of errors in the data regarding consistency and accuracy of participant IDs used to match pre–post surveys and parent/caregiver–adolescent dyads, which is common among datasets collected in routine delivery settings.^17^ First, there were many instances in which it was not possible to link the data provided by parents/caregivers and adolescents from the same family. Second, there were numerous instances in which participants provided information that contradicted expectations regarding programme delivery. For instance, 1752 adolescents provided ‘0’ to every survey question. Third, there were a large number of missing time points/surveys. It is possible that participants who did not complete post-test surveys due to programme dropout or refused to answer certain questions may have experienced worse effects. Likewise, we were unable to examine the potential effect of missing data at the facilitator level due inconsistent data collection by implementing partners. More efficient processes for assigning participant IDs may reduce errors. Improvements in monitoring and evaluation systems would require additional training for data collectors on how to identify inconsistent responses.^40^ It is also possible that those who were dropped from the study due to missing data may have had lower programme engagement than those who were included, which may have resulted in overestimation of intervention effects. Nonetheless, sensitivity analyses using data that only removed duplicate forms showed very similar results to the main analyses. Fourth, the reliance on secondary pre–post data collected from routine service delivery systems limited our ability to draw causal inference of programme effectiveness at scale. Although sensitivity analyses using E-values did not reveal significant unmeasured confounding effects, it may have been more prudent to use the staggered rollout of the programme to randomly select a natural control group. However, embedding more complex study designs within a large-scale initiative would have required additional time and funding to adequately plan with implementing partners.

## Conclusion

This study is the first to examine the large-scale implementation of an evidence-based parenting programme in the Global South. It demonstrates the importance of harnessing real-world data to examine the scale-up of an evidence-based intervention in a low-resource context. Findings suggest that Furaha Teens may have a positive impact on key outcomes associated with VAC when delivered at scale. Although the pre–post design could not establish causality, results suggest that parenting programmes may be effective at reducing violence against adolescent girls at home and school. Results indicating positive effects on reduced experience of IPV and school violence, improved parent/caregiver-adolescent communication about sexual health, and other outcomes are promising. The reduced positive parenting outcomes are concerning, though our interpretation of effects is limited due to the lack of a comparison group. Nonetheless, consistent positive results across parent/caregiver reports and adolescent reports suggest that Furaha Teens maintains its effectiveness when delivered at scale in Tanzania. A forthcoming qualitative analysis conducted as part of the FAIR study will provide further insight into the mechanisms of change experienced by participating families as well as how the quality of delivery by facilitators may have impacted outcomes. In addition, analyses suggest that further support may be needed for more vulnerable children, such as through referrals to other services. Finally, innovative approaches using hybrid or digital formats may reduce the human and financial resources associated with large-scale delivery as well as improve the monitoring and evaluation of programmes at scale.

## Supplementary material

10.1136/bmjgh-2024-015472 online supplemental file 1

10.1136/bmjgh-2024-015472 online supplemental file 2

## Data Availability

Data are available on reasonable request.
