# Supplementary material for: Reducing family and school-based violence at scale: a large-scale pre–post study of a parenting programme delivered to families with adolescent girls in Tanzania
Source: BMJ Glob Health. 2024 Nov 24;9(11):e015472. doi: 10.1136/bmjgh-2024-015472 (PMC11590853; doi:10.1136/bmjgh-2024-015472)
Supplement: online supplemental file 1 [file bmjgh-9-11-s001.pdf]

Reducing family and school-based violence at scale: A large-scale pre-post study of a parenting programme delivered to families with adolescent girls in Tanzania – Supplementary File

Supplementary Figure 1. Distribution Test Flowchart

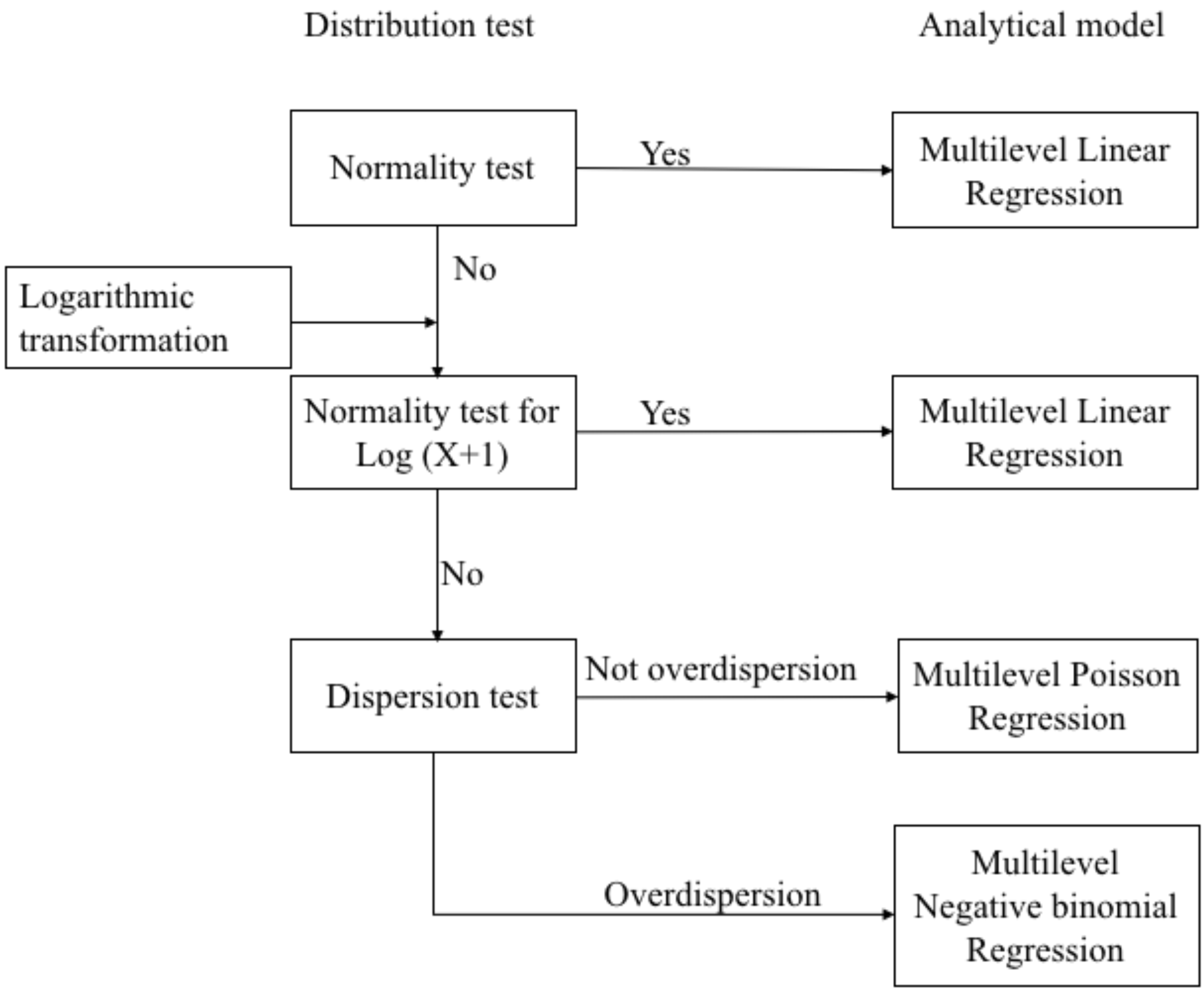

Supplementary Table 1. TiDieR Check-list

Item number	Item	Page
	**BRIEF NAME**	
**1.**	Provide the name or a phrase that describes the intervention.	6
	**WHY**	
**2.**	Describe any rationale, theory, or goal of the elements essential to the intervention.	8-9
	**WHAT**	
**3.**	Materials: Describe any physical or informational materials used in the intervention, including those provided to participants or used in intervention delivery or in training of intervention providers. Provide information on where the materials can be accessed (e.g. online appendix, URL).	8-9
**4.**	Procedures: Describe each of the procedures, activities, and/or processes used in the intervention, including any enabling or support activities.	8-9
	**WHO PROVIDED**	
**5.**	For each category of intervention provider (e.g. psychologist, nursing assistant), describe their expertise, background and any specific training given.	8
	**HOW**	
**6.**	Describe the modes of delivery (e.g. face-to-face or by some other mechanism, such as internet or telephone) of the intervention and whether it was provided individually or in a group.	8-9
	**WHERE**	
**7.**	Describe the type(s) of location(s) where the intervention occurred, including any necessary infrastructure or relevant features.	8
	**WHEN and HOW MUCH**	
**8.**	Describe the number of times the intervention was delivered and over what period of time including the number of sessions, their schedule, and their duration, intensity or dose.	8-9
	**TAILORING**	
**9.**	If the intervention was planned to be personalised, titrated or adapted, then describe what, why, when, and how.	6
	**MODIFICATIONS**	
**10.**[‡]	If the intervention was modified during the course of the study, describe the changes (what, why, when, and how).	N/A

HOW WELL

**11.**	Planned: If intervention adherence or fidelity was assessed, describe how and by whom, and if any strategies were used to maintain or improve fidelity, describe them.	N/A
**12.**‡	Actual: If intervention adherence or fidelity was assessed, describe the extent to which the intervention was delivered as planned.	N/A

Supplementary Table 2. Distribution checks based on caregiver report

	Anderson-Darling normality test		Anderson-Darling normality test (log x+1)		Dispersion test		Distribution
	W	p value	W	p value	Chi-sq	p value	
Overall maltreatment	3214.62	<0.001	2398.79	<0.001	42336.12	1.000	Poisson
Physical abuse	4892.51	<0.001	4684.47	<0.001	33045.52	1.000	Poisson
Psychological abuse	4008.22	<0.001	3598.38	<0.001	33962.32	1.000	Poisson
IPV victimisation	2520.33	<0.001	2593.61	<0.001	10377.84	1.000	Poisson
IPV perpetration	2700.31	<0.001	2747.06	<0.001	10286.34	1.000	Poisson

Supplementary Table 3. Distribution checks based on adolescent report

	Anderson-Darling normality test		Anderson-Darling normality test (log x+1)		Dispersion test		Distribution
	W	p-value	W	p-value	Chi-sq	p-value	
Overall maltreatment	3920.18	<0.001	3018.16	<0.001	42336.12	0.982	Poisson
Physical abuse	5150.58	<0.001	4660.03	<0.001	33045.52	1.000	Poisson
Psychological abuse	4985.51	<0.001	4512.29	<0.001	33962.32	1.000	Poisson
School violence victimisation	1712.23	<0.001	1492.15	<0.001	10377.84	1.000	Poisson

Supplementary Table 4. Reliability checks of pre-post scales

Caregiver-Reported Outcomes	Cronbach's Alpha	Omega	Pearson Correlation
Child maltreatment[1]	$\alpha = 0.65$	$\omega = 0.65$	-
Physical abuse[1]	-	-	$r = 0.20$
Emotional abuse[1]	-	-	$r = 0.24$
Positive parental involvement[2]	$\alpha = 0.95$	$\omega = 0.95$	-
Poor parental supervision[2]	$\alpha = 0.81$	$\omega = 0.81$	-
Child conduct problems[3]	$\alpha = 0.70$	$\omega = 0.75$	-
Parental depression[4]	$\alpha = 0.57$	$\omega = 0.67$	-
Sexual health communication[5]	$\alpha = 0.91$	$\omega = 0.91$	-
Financial insecurity[6]	-	-	$r = 0.79$
Parental support for education[7]	-	-	$r = 0.90$
Parenting stress[8]	-	-	$r = 0.70$
IPV victimisation[9]	-	-	$r = 0.57$
IPV perpetration[9]	-	-	$r = 0.58$
**Adolescent-Reported Outcomes**	**Cronbach's Alpha**	**Omega**	**Pearson Correlation**
Child maltreatment[1]	$\alpha = 0.64$	$\omega = 0.64$	-
Physical abuse[1]	-	-	$r = 0.18$
Emotional abuse[1]	-	-	$r = 0.61$
Positive parental involvement[2]	$\alpha = 0.94$	$\omega = 0.94$	-
Poor parental supervision[2]	$\alpha = 0.77$	$\omega = 0.77$	-
Child conduct problems[3]	$\alpha = 0.68$	$\omega = 0.74$	-
Child emotional problems[3]	$\alpha = 0.90$	$\omega = 0.90$	-
Child depression[4]	$\alpha = 0.69$	$\omega = 0.71$	-
Sexual health communication[5]	$\alpha = 0.90$	$\omega = 0.90$	-
School violence	$\alpha = 0.79$	$\omega = 0.79$	-
Parental support for education[7]	-	-	$r = 0.90$

[1] ISPCAN Child Abuse Screening Tool-Trial; [2] Alabama Parenting Questionnaire; [3] Strengths and Difficulties Questionnaire; [4] Centre for Epidemiological Studies-Depression; [5] Risk Avoidance Planning Scale; [6] Family Financial Coping Scale; [7] Parental Support for School Scale; [8] Parenting Stress Scale; [9] Revised Conflict Tactics Scale Short

Supplementary Table 5. Intraclass Correlation Coefficient (ICC) for Three Level Factors

Random effect	ICC	SE	95% CI
Participant ID	0.70	0.01	0.69, 0.71
Facilitator ID	0.75	0.03	0.69, 0.81
Wave of Implementation	0.12	0.11	-0.11, 0.34

Supplementary Table 6. E-value for parent report variables

**Variable**		**point**	**lower CI**	**upper CI**
Overall maltreatment	RR	0.55	0.54	0.56
	E-value	3.05	NA	2.99
Physical abuse	RR	0.51	0.5	0.53
	E-value	3.3	NA	3.21
Psychological abuse	RR	0.56	0.55	0.57
	E-value	2.98	NA	2.91
IPV victimisation	RR	0.81	0.78	0.84
	E-value	1.79	NA	1.68
IPV perpetration	RR	0.86	0.83	0.9
	E-value	1.59	NA	1.48
Positive parent involvement	RR	0.72	0.7	0.74
	E-value	2.12	NA	2.03
Poor parent supervision	RR	0.77	0.76	0.79
	E-value	1.91	NA	1.86
Parent support of education	RR	0.76	0.74	0.79
	E-value	1.95	NA	1.86
Financial insecurity	RR	0.64	0.63	0.65
	E-value	2.49	NA	2.43
Parenting stress	RR	0.56	0.55	0.57
	E-value	3	NA	2.93
Parenting depression	RR	0.69	0.68	0.7
	E-value	2.25	NA	2.2
Child conduct problems	RR	0.88	0.86	0.89
	E-value	1.54	NA	1.49
Sexual health communication	RR	2.02	1.98	2.05
	E-value	3.45	3.38	NA

Supplementary Table 7. E-value for child report variables

Variable		point	lower CI	upper CI
Overall maltreatment	RR	0.57	0.56	0.58
	E-value	2.92	NA	2.86
Physical abuse	RR	0.56	0.55	0.58
	E-value	2.95	NA	2.87
Psychological abuse	RR	0.55	0.54	0.56
	E-value	3.05	NA	2.96
School violence victimisation	RR	0.84	0.82	0.86
	E-value	1.67	NA	1.58
Positive parent involvement	RR	0.79	0.77	0.81
	E-value	1.84	NA	1.76
Poor parent supervision	RR	0.72	0.71	0.73
	E-value	2.13	NA	2.08
Parent support of education	RR	0.82	0.80	0.84
	E-value	1.74	NA	1.65
Child depression	RR	0.82	0.80	0.83
	E-value	1.75	NA	1.70
Child emotional problems	RR	1.01	0.98	1.04
	E-value	1.12	1.00	NA
Child conduct problems	RR	0.90	0.88	0.92
	E-value	1.46	NA	1.41
Sexual health communication	RR	2.20	2.16	2.24
	E-value	3.82	3.75	NA

Supplementary Table 8. Characteristics of included and excluded participants

Characteristics	Caregivers *n* = 24,863	Adolescents *n* = 24,863	Excluded Caregivers *n* = 5,519	Excluded Adolescents *n* = 3,516
Age, *M* (*SD*)	44.11 (11.82)	11.64 (1.52)	44.75 (11.80)	11.56 (1.51)
Gender: Female, n (%)	16,067 (64.6)	-	3,408 (61.8)	-
Child education level, *M* (*SD*)	4.83 (1.86)	-	4.53 (2.00)	-
Child enrolled in school, n (%)	8,498 (78.2)	-	1,203 (69.7)	-
Currently employed, n (%)	7,503 (30.2)	-	1,474 (26.7)	-
Marital status: Partnered, n (%)	9,273 (85.4)	-	1,501 (86.9)	-
Child biological son/daughter, n (%)	21,432 (86.2)	-	3,303 (85.0)	-
Household struggles to buy food or essentials, n (%)	13,005 (52.3)	10,402 (41.8)	1,879 (48.4)	895 (29.0)
Unwell adult in house, n (%)	2,475 (10.5)	2,000 (8.4)	351 (9.6)	154 (5.3)
Household affected by TB or HIV/AIDS, n (%)	1,725 (6.9)	1,199 (4.8)	306 (7.9)	115 (3.7)
Household affected by alcohol or drugs, n (%)	3,592 (14.4)	3,562 (14.3)	522 (13.4)	226 (7.3)
Household affected by arguments, n (%)	2,857 (11.5)	2,876 (11.6)	389 (10.0)	156 (5.0)
Unwell child in house, n (%)	2,886 (11.6)	2,443 (9.8)	482 (12.4)	189 (6.1)
Disability affects a child in house, n (%)	1,587 (6.4)	948 (3.8)	248 (6.4)	105 (3.4)
Biological parent lives in house, n (%)	20,452 (82.3)	21,418 (86.1)	3,214 (82.7)	2,757 (89.2)
Can read easily, n (%)	14,988 (60.3)	19,218 (77.3)	2,037 (52.4)	2,271 (73.5)
Child single or double orphan, n (%)	4,530 (18.2)	3,764 (15.1)	653 (16.8)	309 (10.0)
Adolescent parenthood, n (%)	-	950 (3.8)	-	76 (2.5)
Sessions attended, *M* (*SD*)	12.75 (1.84)	12.76 (1.83)	-	-
Location implemented: School, n (%)	8181 (77.8)		-	-
Facilitator type: Teacher, n (%)	7588 (72.1)		-	-

Supplementary Table 9. Multilevel regression analysis of caregiver-reported outcomes before merging with adolescent data

	Mean pre	SD pre	Mean post	SD post	beta	SE	p value	IRR	95% lower CI	95% upper CI
Overall maltreatment[1]	2.29	2.72	1.20	1.97	-0.58	0.01	<0.001	0.56	0.55	0.57
Physical abuse[1]	1.06	1.46	0.53	1.08	-0.64	0.01	<0.001	0.53	0.51	0.54
Psychological abuse[1]	1.23	1.59	0.67	1.14	-0.57	0.01	<0.001	0.57	0.55	0.58
IPV experience (female-only)[2]	1.07	1.76	0.73	1.45	-0.26	0.02	<0.001	0.78	0.74	0.81
IPV perpetration (male-only)[2]	0.86	1.67	0.76	1.57	0.03	0.03	0.240	1.03	0.98	1.09
Positive parent involvement[3]	4.60	4.04	3.28	3.82	-0.25	0.01	<0.001	NA	NA	NA
Poor parent supervision[3]	1.26	1.93	0.77	1.53	-0.49	0.02	<0.001	NA	NA	NA
Parent support of education[4]	5.04	2.79	4.28	2.64	-0.69	0.03	<0.001	NA	NA	NA
Financial insecurity[5]	2.85	2.20	1.93	2.00	-0.91	0.02	<0.001	NA	NA	NA
Parenting stress[6]	3.23	2.60	1.84	2.26	-1.38	0.02	<0.001	NA	NA	NA
Parenting depression[7]	3.89	1.43	3.35	1.39	-0.54	0.01	<0.001	NA	NA	NA
Child conduct problems[8]	1.72	1.76	1.49	1.77	-0.20	0.01	<0.001	NA	NA	NA
Sexual health communication[9]	2.20	1.99	3.65	2.40	1.45	0.02	<0.001	NA	NA	NA

Note: Positive parent involvement and parent support of education only measured during Wave 2 of programme delivery; [1] ISPCAN Child Abuse Screening Tool-Trial; [2] Revised Conflict Tactics Scale Short; [3] Alabama Parenting Questionnaire; [4] Parental Support for School Scale; [5] Family Financial Coping Scale; [6] Parenting Stress Scale; [7] Centre for Epidemiological Studies-Depression Short-Form; [8] Strengths and Difficulties Questionnaire Conduct Problems Subscale; [9] Risk Avoidance Planning Scale

Supplementary Table 10. Multilevel regression analysis of adolescent-reported outcomes before merging with caregiver data

	Mean pre	SD pre	Mean post	SD post	beta	SE	p-value	IRR	95% lower CI	95% upper CI
Overall maltreatment[1]	2.17	2.83	1.14	2.16	-0.55	0.01	<0.001	0.57	0.57	0.58
Physical abuse[1]	1.06	1.57	0.57	1.22	-0.56	0.01	<0.001	0.57	0.56	0.58
Psychological abuse[1]	1.10	1.60	0.57	1.18	-0.59	0.01	<0.001	0.55	0.54	0.56
School violence experience[2]	2.24	3.14	1.51	2.76	-0.20	0.01	<0.001	0.82	0.80	0.84
Positive parent involvement[3]	4.97	3.91	4.08	3.67	-0.81	0.05	<0.001	NA	NA	NA
Poor parent supervision[3]	1.32	1.93	0.75	1.42	-0.57	0.01	<0.001	NA	NA	NA
Parent support of education[4]	2.78	2.72	2.22	2.70	-0.48	0.03	<0.001	NA	NA	NA
Child depression[5]	2.31	2.06	1.87	1.98	-0.43	0.02	<0.001	NA	NA	NA
Child emotional problems[6]	1.54	1.98	1.55	2.04	0.02	0.03	0.579	NA	NA	NA
Child conduct problems[7]	1.56	1.69	1.39	1.70	-0.14	0.01	<0.001	NA	NA	NA
Sexual health communication[8]	1.94	1.97	3.43	2.44	1.50	0.02	<0.001	NA	NA	NA

Note: Positive parent involvement and parent support of education only measured during Wave 2 of programme delivery; [1] ISPCAN Child Abuse Screening Tool-Trial; [2] Locally Developed School Violence Scale; [3] Alabama Parenting Questionnaire; [4] Parental Support for School Scale; [5] Centre for Epidemiological Studies-Depression; [6] Strengths and Difficulties Questionnaire; [7] Strengths and Difficulties Questionnaire; [8] Risk Avoidance Planning Scale
