# Supplementary material for: Reducing family and school-based violence at scale: a large-scale pre–post study of a parenting programme delivered to families with adolescent girls in Tanzania
Source: BMJ Glob Health. 2024 Nov 24;9(11):e015472. doi: 10.1136/bmjgh-2024-015472 (PMC11590853; doi:10.1136/bmjgh-2024-015472)
Supplement: online supplemental file 2 [file bmjgh-9-11-s002.pdf]

This study aimed to address pressing local priorities in Tanzania, specifically the reduction of violence against children (VAC) and its association with adolescent HIV risk. This study represents a collaborative effort between the Tanzania National Institute for Medical ResearchNIMR, Oxford University, Cardiff University, Clowns Without Borders South Africa, and Pact Tanzania. Our goal was to foster a transparent, collaborative research environment that not only contributed to academic knowledge but also supported meaningful change in Tanzanian communities. Equal contributions were made across the research team, underscoring a commitment to equity and inclusivity. Local researchers from the Tanzania National Institute for Medical Research (NIMR) and Pact Tanzania were instrumental in conceptualizsing the study, ensuring that it was contextually relevant and aligned with national policy needs while respecting local autonomy and expertise. The majority of funding from the Network of European Foundations Evaluation Fund was allocated to local research efforts and capacity building within NIMR and Pact Tanzania, facilitating data collection and analysis. Field staff, who played crucial roles in data collection, were duly acknowledged, and all partners had equal access to the study data via secure servers. Reflections on positionality revealed potential power imbalances between international and local researchers. To address these, we adopted a collaborative approach throughout the research process, ensuring that local voices were heard in decision-making. Regular meetings facilitated open discussions about ongoing implementation challenges and allowed local researchers to contribute meaningfully to data interpretation. Co-principal investigators, JW and JML worked together to ensure equitable leadership. Key decisions—including the selection of outcome measures, data analysis, and result interpretation—were made collaboratively. Local researchers from Pact Tanzania and the Tanzania National Institute for Medical ResearchNIMR played critical roles in ensuring contextual relevance and maintaining ethical standards, especially during large-scale data collection amidst the COVID-19 pandemic. The authorship team was gender- balanced, recognizsing the contributions of both male and female researchers. Training opportunities extended to methodological skills and infrastructure improvements, enhancing local capacity for future research. Ethical governance procedures, including safeguarding measures, ensured the protection of participants and researchers, with ethical approvals obtained from NIMR and Oxford University. Lastly, findings have been disseminated through local workshops and policy briefs to address community needs directly.
